# Associations Between Hepatitis B Virus Infection and Risk of All Cancer Types

**DOI:** 10.1001/jamanetworkopen.2019.5718

**Published:** 2019-06-14

**Authors:** Ci Song, Jun Lv, Yao Liu, Jian Guo Chen, Zijun Ge, Jian Zhu, Juncheng Dai, Ling-Bin Du, Canqing Yu, Yu Guo, Zheng Bian, Ling Yang, Yiping Chen, Zhengming Chen, Jibin Liu, Jie Jiang, Liguo Zhu, Xiangjun Zhai, Yue Jiang, Hongxia Ma, Guangfu Jin, Hongbing Shen, Liming Li, Zhibin Hu

**Affiliations:** 1 Department of Epidemiology and Biostatistics, Jiangsu Key Lab of Cancer Biomarkers, Prevention and Treatment, Collaborative Innovation Center for Cancer Personalized Medicine, School of Public Health, Nanjing Medical University, Nanjing, China; 2 Department of Epidemiology and Biostatistics, School of Public Health, Peking University Health Science Center, Beijing, China; 3 Pathology Center and Department of Pathology, Soochow University, Suzhou, China; 4 Qidong Liver Cancer Institute, The First People’s Hospital of Qidong, Qidong, China; 5 Zhejiang Cancer Center, Zhejiang Cancer Hospital, Hangzhou, China; 6 Chinese Academy of Medical Sciences, Beijing, China; 7 Clinical Trial Service Unit and Epidemiological Studies Unit, Nuffield Department of Population Health, University of Oxford, United Kingdom; 8 Department of Hepatobiliary Surgery, Nantong Tumor Hospital, Nantong, China; 9 Jiangsu Province Center for Disease Prevention and Control, Nanjing, China

## Abstract

**Importance:**

Hepatitis B virus (HBV) has been identified as a major risk factor for hepatocellular carcinoma. However, the associations between HBV infection and other cancer types are not well understood.

**Objective:**

To assess the associations between chronic HBV infection and risk of all cancer types.

**Design, Setting, and Participants:**

This population-based study involved 3 cohorts in China. The China Kadoorie Biobank (CKB) prospective cohort study, conducted between June 2004 and July 2008, used a dipstick assay for detection of serum hepatitis B surface antigen (HBsAg) among 496 732 participants to determine the association between HBV infection and risk of all cancer types. Two cohort studies were used to validate the associations by applying more precise serum HBsAg detection assays: the Qidong cohort (37 336 participants enrolled from November 2007 to April 2011) and the Changzhou nested case-control study (17 723 participants enrolled from June 2004 to September 2005). A total of 97 samples of stomach cancer tissues, 10 samples of pancreatic cancer tissues, and 9 samples of lung cancer tissues were included to assess the presence of HBV replication and expression. Statistical analysis was performed from December 2016 to October 2018.

**Exposures:**

Serum HBsAg status in the population-based stage and HBV DNA status, the expression of hepatitis B X protein, and hepatitis B core antibody (anti-HBc) in the tissue-based stage.

**Main Outcomes and Measures:**

Incidence of all cancer types during follow-up.

**Results:**

In the CKB cohort, the mean (SD) age of the 496 732 participants was 51.5 (10.7) years; 59.0% of the participants were women. After 4.4 million person-years of follow-up, participants who were HBsAg seropositive (n = 15 355) had a higher risk of hepatocellular carcinoma (hazard ratio [HR], 15.77; 95% CI, 14.15-17.57), stomach cancer (HR, 1.41; 95% CI, 1.11-1.80), colorectal cancer (HR, 1.42; 95% CI, 1.12-1.81), oral cancer (HR, 1.58; 95% CI, 1.01-2.49), pancreatic cancer (HR, 1.65; 95% CI, 1.03-2.65), and lymphoma (HR, 2.10; 95% CI, 1.34-3.31) when compared with participants who were HBsAg seronegative (n = 481 377). Because of the limitation of sample size, only associations of HBV infection with hepatocellular carcinoma and stomach cancer were validated in the Qidong cohort (hepatocellular carcinoma: HR, 17.51; 95% CI, 13.86-22.11; stomach cancer: HR, 2.02; 95% CI, 1.24-3.29); the Changzhou nested case-control study validated only an association between HBV infection and stomach cancer (odds ratio, 1.76; 95% CI, 1.04-2.98). Moreover, among 22 participants with stomach cancer from the Qidong cohort who were anti-HBc seropositive, 12 samples (54.5%) of cancer tissues were HBV DNA positive, while among 25 participants with stomach cancer who were anti-HBc seronegative, no HBV DNA was detected. The same negative and positive rate was observed in the validation set from Zhejiang Tumor Hospital (19 of 35 samples [54.3%] were HBV DNA positive). Moreover, among the 8 patients with stomach cancer from the Qidong cohort who were anti-HBc seropositive, anti-HBc and hepatitis B X protein were expressed in all of their stomach cancer tissue samples. The same phenomenon was observed in the patients with pancreatic cancer but not in the patients with lung cancer, which was consistent with the population-based results of the CKB cohort.

**Conclusions and Relevance:**

This study found that HBV infection was also associated with the risk of nonliver cancer, especially digestive system cancers among adults in China.

## Key Points

**Question:**

Is chronic hepatitis B virus infection associated with a higher risk of any cancer type when compared with individuals without hepatitis B virus?

**Findings:**

This population-based cohort study of 496 732 Chinese individuals found that hepatitis B surface antigen seropositivity was associated with the risk of hepatocellular carcinoma, stomach cancer, colorectal cancer, oral cancer, pancreatic cancer, and lymphoma. The associations were validated in an independent population and tissue-based studies.

**Meaning:**

Chronic hepatitis B virus infection was associated with nonliver cancer, especially digestive system cancers.

## Teaser

This population-based cohort study examines the associations between chronic hepatitis B virus infection and risk of all cancer types among Chinese adults.

## Introduction

Hepatitis B virus (HBV) infection is one of the most serious and prevalent health conditions, affecting more than 2 billion people worldwide.^1^ Hepatitis B virus has been implicated in the cause of up to 80% of cases of hepatocellular carcinoma (HCC), which frequently occurs in Chinese and African populations.^2^ The virus optimizes its life cycle to allow for long-term persistence in liver tissue by establishing a plasmid-like covalently closed circular DNA (cccDNA) form.^3^ Chronic HBV infection persisting in liver tissue is associated with increased chronic oxidative damage in hepatocytes, immune-mediated inflammation of the liver, and development of cancer.^4^

A few clinical case studies detected HBV in several types of nonliver tissues, suggesting a potential role of HBV in the oncogenesis of nonliver cancers.^5,6,7^ Few population-based prospective studies have observed associations between chronic HBV infection and various nonliver cancers, but these findings were inconsistent.^8,9,10,11,12^ The lack of detailed individual information led to minimal control of potential confounding. Hospital-based identification of participants might also overestimate the incidence of nonliver cancer in patients with HBV. Furthermore, previous population-based studies used the level of HBV infection markers in peripheral blood, which represents the degree of virus replication in the liver instead of that in the nonliver tissues.^13,14^ Therefore, whether the observed association between HBV infection and nonliver cancers was owing to the general influence of the virus on the immune system or specific attacks on the nonliver histocytes remains unclear.

In the present study, we aimed to examine the associations between chronic HBV infection and risk of all types of cancer using 3 substudies with complementary advantages. We first analyzed the associations between HBV infection and all types of cancer in the China Kadoorie Biobank (CKB) study of 496 732 adults, in which HBV infection was detected using an onsite hepatitis B surface antigen (HBsAg) rapid test (substudy 1). To minimize the potential influence of infection misclassification on effect estimation, we replicated the association analysis in the Qidong prospective cohort and a nested case-control study established within the Changzhou cohort; both of these cohorts used more sensitive detection assays for HBsAg (substudy 2). Finally, we examined whether HBV replication and expression existed in the cancer tissues of the patients with stomach cancer, pancreatic cancer, and lung cancer (substudy 3).

## Methods

### Study Population

#### Substudy 1

The CKB study is a prospective cohort study of more than 0.5 million adults from 10 geographically diverse areas across China, in which 512 891 participants aged 30 to 79 years were enrolled between June 2004 and July 2008.^15,16^ At baseline, trained health workers administered a laptop-based questionnaire on sociodemographic characteristics, lifestyle behaviors, and personal and family medical histories, and measured anthropometric indexes. Incident cancer cases since the participants’ enrollment into the study were identified by means of linkage with local disease and death registries, the national health insurance system, and by active follow-up.^16^ Nearly all of the CKB population is now covered by the health insurance system, which records details of all hospitalized events (with *International Classification of Diseases and Related Health Problems, Tenth Revision* codes) and coded examination and treatment procedures. The first reported cancer type was regarded as the primary cancer among the participants who had reported 2 or more cancer sites. The study protocol was approved by the ethics review committee of the Chinese Centers for Disease Control and Prevention (Beijing, China) and the Oxford Tropical Research Ethics Committee, University of Oxford (Oxford, United Kingdom). Institutional review boards at all participating centers approved the study, and all participants gave written informed consent.^16^ This study followed the Strengthening the Reporting of Observational Studies in Epidemiology (STROBE) reporting guidelines.

We excluded 11 733 participants who had missing data or unclear HBsAg test results, 2522 participants with cancer at baseline, and 1904 participants who developed cancer during the first year of follow-up. A total of 496 732 participants were included in the final analysis (Figure).

**Figure.**
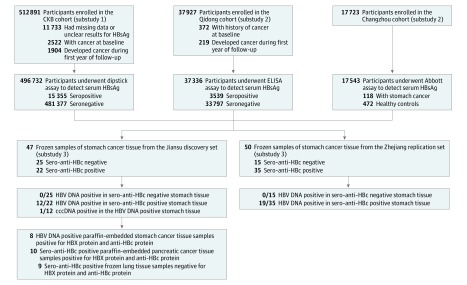
Flowchart of the Association Study Between Hepatitis B Virus (HBV) Infection and Extrahepatic Cancer Substudies 1 and 2 were the population-based exploration. Substudy 3 examined HBV replication and expression in extrahepatic tissues. anti-HBc indicates hepatitis B core antibody; cccDNA, covalently closed circular DNA; CKB, China Kadoorie Biobank; ELISA, enzyme-linked immunosorbent assay; HBsAg, hepatitis B surface antigen; and HBX, hepatitis B X.

#### Substudy 2

##### The Qidong Prospective Cohort

A population-based cohort study was established from November 2007 to April 2011 in Qidong County, Jiangsu Province. In 6 randomly selected villages, inhabitants aged 30 to 70 years who had been living in their current residence for at least 5 years were eligible to participate. A total of 37 927 participants provided written informed consent for the interview and blood collection for serologic assays. Only baseline age and sex were collected for participants who were HBsAg negative. Cancer incidence among participants was obtained from the Qidong Cancer Registry, which is a population-based registry system established in 1972 by the local health authority. The Qidong Cancer Registry uses both active and passive methods for cancer data collection. Further details of these methods have been described previously.^17^ The first reported cancer type was regarded as the primary cancer among the participants who had reported 2 or more cancer sites. In the present analysis, we excluded participants who reported a history of cancer at baseline (n = 372) and those who developed cancer during the first year of follow-up (n = 219), leaving 37 336 participants for analysis (Figure).

##### The Changzhou Nested Case-Control Study

A population-based cohort was established between June 2004 and September 2005 in the Wujin District in Changzhou City, Jiangsu Province. Detailed descriptions of the study have been reported previously.^18^ Overall, 17 723 participants 35 years or older who had been living in their current residence for at least 5 years were eligible to participate. All participants who provided written informed consent were interviewed using a structured questionnaire to collect sociodemographic characteristics, lifestyle behaviors, and personal and family medical histories; anthropometric indexes were also measured. The first follow-up survey, conducted from April 2008 to October 2009, and the second follow-up survey, conducted from April 2012 to November 2013, resulted in the identification of 141 new cases of stomach cancer; 118 of these patients provided sufficient blood samples. We then performed a nested case-control study in which 118 patients with stomach cancer were matched with 472 healthy controls (1:4) on the basis of age and sex (Figure).

#### Substudy 3

The surgically resected tissues from patients with primary stomach cancer were collected from (1) Nantong Tumor Hospital (Nantong, Jiangsu, China) and First Affiliated Hospital of Suzhou University (Suzhou, Jiangsu, China) between January 2014 and December 2015 (n = 47); and from (2) Zhejiang Tumor Hospital (Hangzhou, Zhejiang, China) between June 2011 and December 2012 (Figure). The samples of cancer tissues were frozen in RNAlater Stabilization Solution (Thermo Fisher Scientific) immediately after excision and stored at –80 °C until DNA isolation.

The paraffin-embedded tissues from patients with primary pancreatic cancer were collected from the First Affiliated Hospital of Nanjing Medical University (Nanjing, Jiangsu, China) between January 2015 and December 2016 (n = 10). Frozen tissues from patients with primary lung cancer were collected from Jiangsu Tumor Hospital (Nanjing, Jiangsu, China) between January 2015 and December 2016 (n = 9) (Figure).

### Assessment of HBV Infection, Replication, and Expression

#### Serologic Testing

In substudy 1, CKB participants were tested for baseline serum HBsAg using on-site rapid test strips (ACON Laboratories), a convenient and repeatable method but with low sensitivity for lower sero-HBsAg levels; results using these test strips were negative, positive, or unclear. In substudy 2, Qidong participants were tested using enzyme-linked immunosorbent assay (Kehua Bio-Engineering Co Ltd), and Changzhou participants were tested with Architect HBsAg QT assay kits (Abbott Laboratories), which have a lower limit of detection of 0.05 IU/mL. The enzyme-linked immunosorbent assay is one of the most common methods for screening HBsAg, with a moderate price and better accuracy. The chemiluminescence-based Abbott assay is the most sensitive method. In substudy 3, all surgically resected tissue samples collected from patients with cancer were tested for serum HBsAg and hepatitis B core antibody (anti-HBc) using the enzyme-linked immunosorbent assay.

#### Detection of HBV DNA and cccDNA in Stomach Cancer Tissue

In substudy 3, genomic DNA was extracted from all 97 frozen tissue samples of stomach cancer using the QIAamp DNA Mini Kit (QIAGEN GmbH). Hepatitis B virus replication and cccDNA were identified by polymerase chain reaction using a modified version of the process described by Laras et al.^19^ Polymerase chain reaction amplification was performed using Phusion High-Fidelity DNA polymerase (Thermo Fisher Scientific) to detect HBV DNA and cccDNA in tissue samples from patients with stomach cancer. Hepatitis B v DNA was detected using 3′ antisense primer BC1 (5′-GGAAAGAAGTCAGAAGGCAA nt1974-1955) and 5′ primers PGP (5′-CACCTCTGCCTAATCATC nt1826-1843). Tissues from Jiangsu patients who were positive for HBV DNA were then tested for cccDNA using 3′ antisense primer BC1 (5′-GGAAAGAAGTCAGAAGGCAA nt1974-1955) and 5′ primers CCC (5′-GTGCCTTCTCATCTGCCGG nt1555-1573). Meanwhile, we also amplified the DNA from 1 HCC tissue sample to serve as a positive control (eFigure 1 in the Supplement); in that sample, the serum HBV level was higher than 5 × 10^6^ copies/mL. The polymerase chain reaction–amplified products underwent electrophoresis on 2% agarose gel, stained with ethidium bromide and visualized under UV light (eFigure 1 in the Supplement). The product was then sequenced and aligned with the reference sequences, which had been aligned using 100 full-length HBV sequences (genotype B and C) obtained from GenBank.^20^

#### Detection of HBV Protein Expression in Cancer Tissue Using Immunohistochemistry Test

In substudy 3, immunohistochemical staining for hepatitis B X (HBX) protein (ab235) and anti-HBc protein (ab8638) (Abcam) was then examined on 4-μm formalin-fixed paraffin-embedded sections of cancer tissue from the 8 patients with stomach cancer and 10 patients with pancreatic cancer, as well as on 4-μm frozen sections of cancer tissue from 9 patients with lung cancer. Anti-HBc represents the HBV virus replication level in cancer tissues; HBX protein acts as a potent transactivator and helps in tumor progression.^21^ The antibodies were diluted 1:100 with an antigen retrieval buffer. The antigen retrieval buffers tested were sodium citrate (pH, 6.0). Conditions for the antibodies were optimized in the laboratory according to the manufacturer’s instructions. An experienced gastrointestinal pathologist (Y.L.) reviewed all specimens.

### Statistical Analysis

Statistical analysis was performed from December 2016 to October 2018. All cancer cases were documented according to the *International Classification of Diseases and Related Health Problems, Tenth Revision*, by coders who were unaware of the baseline characteristics of the study participants (eTable 1 in the Supplement). In substudy 1 of the CKB cohort, we estimated age-, sex-, and region-standardized incidence rates of cancer by the direct standardized method using the 2010 Chinese Census as the standard population. Person-years were calculated as the time from baseline to the date of cancer diagnosis, death, loss to follow-up, or December 31, 2015, whichever occurred first. We used Cox proportional hazards regression to estimate the hazard ratios (HRs) and their 95% CIs with adjustment for baseline covariates (age, sex, region, educational level, household income, marital status, smoking status, alcohol consumption, family cancer history, and body mass index). In substudy 2 of the Qidong cohort, person-years were calculated as the time from baseline to the date of cancer diagnosis, death, loss to follow-up, or December 31, 2015. The Cox proportional hazards regression model was adjusted only for age and sex. For the analysis of the Changzhou nested case-control study, we performed logistic regression analyses to calculate odds ratios and their 95% CIs. The significance level was set at *P* < .05, and all *P* values were 2-sided. Data analyses were performed using R, version 3.0.2 (R Project for Statistical Computing).

## Results

### Substudy 1: Association of HBV Infection With Overall and Site-Specific Cancer in CKB Cohort

The mean (SD) age of the 496 732 participants was 51.5 (10.7) years; 59.0% of the participants were women, 41.0% were men, and 44.5% were from urban areas (eTable 2 in the Supplement). The prevalence of HBsAg positivity was 3.1% (n = 15 355). Participants who were HBsAg positive were more likely to be younger, male, and urban residents. During 4.4 million person-years of follow-up, there were 20 891 incident cases of cancer (eTable 3 in the Supplement). Multivariable-adjusted analyses showed that HBsAg seropositivity was associated with an increased risk of overall cancer incidence (HR, 2.18; 95% CI, 2.05-2.32) (Table 1). We checked for nonproportional hazards by testing for a nonzero slope in the scaled Schoenfeld residuals over time. Hepatitis B surface antigen seropositivity was also associated with the risks of several site-specific cancers, including HCC (HR, 15.77; 95% CI, 14.15-17.57), stomach cancer (HR, 1.41; 95% CI, 1.11-1.80), colorectal cancer (HR, 1.42; 95% CI, 1.12-1.81), oral cavity cancer (HR, 1.58; 95% CI, 1.01-2.49), lymphoma (HR, 2.10; 95% CI, 1.34-3.31), and pancreatic cancer (HR, 1.65; 95% CI, 1.03-2.65). There were no statistically significant associations between HBsAg seropositivity and other cancer sites. Hepatitis C core antibody was not detected in the study population; however, the prevalence of hepatitis C core antibody was low in China (0.43%).^22^

**Table 1.** Adjusted Hazard Ratios for Overall Cancer and Site-Specific Cancers According to HBsAg Status in the CKB Cohort

Cancer Site	HBsAg Seropositive (n = 15 355)		HBsAg Seronegative (n = 481 377)		Adjusted Hazard Ratio (95% CI)	*P* Value
	Cases, No.	Incidence Rate, No./1000 PYs	Cases, No.	Incidence Rate, No./1000 PYs		
Overall cancer	1107	8.12	19 784	4.56	2.18 (2.05-2.32)	<.001
Liver (HCC)	514	3.77	1261	0.29	15.77 (14.15-17.57)	<.001
Lung	97	0.71	3750	0.86	1.07 (0.87-1.31)	.54
Stomach	78	0.57	2079	0.48	1.41 (1.11-1.80)	.005
Colorectum	70	0.51	2068	0.48	1.42 (1.12-1.81)	.004
Breast	44	0.32	1650	0.38	0.86 (0.71-1.29)	.78
Esophagus	35	0.26	1554	0.36	0.87 (0.62-1.23)	.43
Oral cavity	20	0.15	395	0.09	1.58 (1.01-2.49)	.05
Lymphoma	20	0.15	349	0.08	2.10 (1.34-3.31)	.001
Pancreas	18	0.13	486	0.11	1.65 (1.03-2.65)	.04
Cervix	15	0.11	684	0.16	0.79 (0.47-1.35)	.39
Leukemia	13	0.10	417	0.10	1.14 (0.66-1.99)	.64
Bladder	12	0.09	332	0.08	1.43 (0.78-2.62)	.25
Ovary	8	0.06	255	0.06	1.12 (0.55-2.27)	.76
Endometrium	7	0.05	266	0.06	0.99 (0.46-2.10)	.97
Prostate	5	0.04	247	0.06	1.02 (0.42-2.49)	.96

Abbreviations: CKB, China Kadoorie Biobank; HBsAg, hepatitis B surface antigen; HCC, hepatocellular carcinoma; PYs, person-years.

### Substudy 2: Association of HBV Infection With Stomach Cancer in Qidong Cohort and Changzhou Nested Case-Control Study

Of the 37 336 participants from the Qidong cohort, 9.5% were HBsAg seropositive. During 255 752 person-years of follow-up, there were 1386 incident cases of cancer documented. Age-adjusted and sex-adjusted Cox proportional hazards regression analysis showed that HBsAg seropositivity was also associated with an increased risk of overall cancer (HR, 3.39; 95% CI, 2.95-3.89) (Table 2). Similarly, the risks of developing HCC (HR, 17.51; 95% CI, 13.86-22.11) and stomach cancer (HR, 2.02; 95% CI, 1.24-3.29) were higher in participants who were HBsAg seropositive than in those who were HBsAg seronegative. In the study population, there were only 5 cases of colorectal cancer among participants who were HBsAg positive, and no cases of oral cancer, pancreatic cancer, or lymphoma reported. After pooling all the other sites of cancer, there was no association observed between HBsAg status and cancer risk. In the Changzhou nested case-control study, we also found an association of HBsAg seropositivity with the risk of stomach cancer (odds ratio, 1.76; 95% CI, 1.04-2.98) (eTable 4 in the Supplement).

**Table 2.** Adjusted Hazard Ratios for Overall Cancer and Site-Specific Cancers According to HBsAg Status in the Qidong Cohort

Cancer Sites	HBsAg Seropositive (n = 3539)		HBsAg Seronegative (n = 33 797)		Adjusted Hazard Ratio (95% CI)	*P* Value
	Cases, No.	Incidence Rate, No./1000 PYs	Cases, No.	Incidence Rate, No./1000 PYs		
Overall cancer	267	11.17	900	3.88	3.39 (2.95-3.89)	<.001
Liver (HCC)	189	7.91	117	0.50	17.51 (13.86-22.11)	<.001
Lung	21	0.88	202	0.87	1.31 (0.84-2.06)	.24
Stomach	19	0.79	109	0.47	2.02 (1.24-3.29)	.005
Breast	10	0.42	62	0.27	1.71 (0.87-3.33)	.12
Other	41	1.71	451	1.95	0.78 (0.53-1.15)	.21

Abbreviations: HBsAg, hepatitis B surface antigen; HCC, hepatocellular carcinoma; PYs, person-years.

### Substudy 3: HBV Replication and Expression in Cancer Tissues

Patients from both Jiangsu and Zhejiang with stomach cancer showed similar distributions of serum anti-HBc and HBV DNA in the stomach cancer tissues (eTable 5 in the Supplement). Among the patients in the Jiangsu cohort, 46.8% of tissue samples (22 of 47) were anti-HBc seropositive; among the Zhejiang cohort, 70.0% of tissue samples (35 of 50) were anti-HBc seropositive. Furthermore, among patients with stomach cancer who were anti-HBc seropositive, the prevalence of HBV DNA positivity in cancer tissues was 54.5% in the Jiangsu cohort (12 of 22) and 54.3% in the Zhejiang cohort (19 of 35). In contrast, no HBV DNA was detected in the samples from patients with stomach cancer who were anti-HBc seronegative. Next, cccDNA amplicons from the 12 patients with stomach cancer in the Jiangsu cohort who were HBV DNA positive were sequenced. After the polymerase chain reaction products were sequenced and aligned, cccDNA was found in 1 tissue sample with positive HBV DNA (eFigure 1 in the Supplement), indicating that HBV could actively replicate in stomach tissue but could not induce HBsAg positivity in the serum.

Moreover, of the 12 patients with stomach cancer in the Jiangsu cohort who were HBV DNA positive, 8 formalin-fixed paraffin-embedded cancer tissue sections were collected to examine HBX protein and anti-HBc protein expression using immunohistochemical staining. All the collected tissue samples were anti-HBc seropositive. Hepatitis B X protein and anti-HBc protein were highly expressed in the stomach cancer cells of all the tissue sections from individuals with stomach cancer, but they were minimally expressed in the normal parts of the specimens (eFigure 2 and eTable 6 in the Supplement). The same phenomenon was observed in all of the tissue sections from patients with pancreatic cancer, the representative HBV-related cancer identified in the CKB cohort. However, we did not find the HBX protein or anti-HBc protein expressed in the lung cancer cells or normal lung epithelial cells of any of the tissue sections from patients with lung cancer, which was also consistent with the findings from the CKB cohort.

## Discussion

In a large prospective Chinese cohort of 496 732 adults, we found that participants who were HBsAg seropositive were at an increased risk of developing HCC and several nonliver cancers, including stomach cancer, oral cancer, colorectal cancer, pancreatic cancer, and lymphoma. The association between HBsAg seropositivity and stomach cancer was further replicated in 2 other small Chinese studies, in which more sensitive assays for HBsAg detection were used. Our tissue-based experiments validated the occurrence of HBV expression in cancer cells located in the stomach and pancreas, but not in lung cancer cells.

### Comparison With Other Studies and Potential Mechanism of Action

Hepatitis B virus infection has been commonly recognized as a risk factor for HCC.^23,24,25^ Our results showed that the risk for HCC in individuals who are seropositive for HBsAg is more than 15 times higher than that in individuals who are not infected with HBV. A few prospective studies have examined the association between HBV infection and nonliver cancers.^8,9,10,11,12^ Overall, most previous studies revealed that HBV was associated with pancreatic cancer (HR, 5.73; and HR, 2.61)^8,12^ and lymphoma (HR, 2.80; HR, 4.89; and HR, 2.10).^9,11,12^ However, a European cohort^10^ proposed that rates of pancreatic cancer and lymphoma were not higher in the HBV-infected cohort compared with individuals not infected with HBV. Except for pancreatic cancer and lymphoma, 1 study^11^ also found that 6 other cancer sites were at an increased risk among individuals with HBV: cancers of the upper aerodigestive tract, lung, kidney, skin (squamous cell carcinoma), thyroid gland, and leukemia; another study found that individuals with HBV were at higher risk for colorectal cancer, gallbladder cancer, kidney cancer, and ovarian cancer.^12^ The reported HBV-related nonliver cancer varied among the previous studies. In our present substudy 1, we observed that HBV was associated with pancreatic cancer (HR, 1.65) and lymphoma (HR, 2.10), which was consistent with previous studies. However, an association between HBV infection and cancers of the upper aerodigestive tract, lung, kidney, skin (squamous cell carcinoma), thyroid gland, leukemia, gallbladder, and ovaries was not observed. Furthermore, we observed that chronic HBV infection was associated with an increased risk of stomach cancer, which was further validated in substudy 2. This finding was consistent with a previous Chinese case-control study,^26^ but not in the Swedish study in which only 7 cases of stomach cancer were reported among patients with HBV infection.^12^ This discrepancy may be owing to the different prevalence of HBV infection and stomach cancer.

Except for HCC and stomach cancer, our study among the CKB cohort also found an association between HBV infection and other cancer types, including oral cancer, colorectal cancer, pancreatic cancer, and lymphoma. However, this association was not observed for lung cancer and leukemia, among others. To validate the results found in the CKB study, we detected HBV expression in pancreatic cancer and lung cancer tissues. Eventually, anti-HBc protein and HBX protein were expressed in pancreatic cancer cells, but not in lung cancer cells, which was consistent with the results in the CKB cohort. This specificity supports a unique underlying mechanism of action rather than a general influence of the immune system.

Some studies have suggested that HBV is a hepatotropic virus, targeting HCC and replicating in hepatocytes.^27,28^ In our study, the detection of HBX protein and anti-HBc protein expression in stomach cancer and pancreatic cancer tissues eliminated potential HBV contamination from blood, which indicated HBV replication and expression. We observed that, in most patients with cancer, HBX protein expression and anti-HBc protein expression were higher in cancer cells than in healthy parts of the specimens. It is possible that HBV is harbored in nonliver cells, which may induce local inflammation. Chronic inflammation induced by HBV infection might play a role in the development of cancer; the viral oncogenic HBX protein may play a direct role in the development of cancer. However, we found a lower association between HBV infection and nonliver cancer when compared with HCC. Although we had confirmed HBV replication and expression in stomach cancer and pancreatic cancer, the immunohistochemistry results suggested that HBX protein and anti-HBc protein were expressed only in the cytoplasm but not the nucleolus; the low rate of detectable cccDNA also indicated that HBV may inactively replicate in nonliver tissues. However, further functional tests are needed to explain the potential mechanism of action.

### Strengths and Limitations

This study provides compelling evidence for the association between HBV infection and all types of cancer. Strengths of the CKB study included its prospective design, the inclusion of a geographically widespread Chinese population living in urban and rural areas, and careful adjustment for potential confounders. A large number of cancer cases allowed us to examine the association between HBV infection and various nonliver cancers. To avoid potential reverse causality bias, we also excluded participants who developed cancer during the first year of follow-up. Considering the low sensitivity of the on-site HBsAg rapid test used in the CKB cohort, we replicated the analysis in 2 other studies using more sensitive detection assays. The finding for stomach cancer was similar, suggesting that potential attenuation of the effect estimates owing to HBV infection misclassification was not significant in the CKB cohort. Furthermore, the trend among different statuses observed in our CKB cohort was consistent with previous reports^29,30,31^ (participants who were younger, married and living in coastal areas were more prone to a higher HBsAg-positive rate) and illustrated the accuracy and specificity of the sero-HBsAg test strips. Moreover, the tissue-based test was performed to help understand the potential mechanism of action underlying the association we observed in the population.

Some limitations also warrant mention. The rate of HBsAg positivity among the CKB cohort was 3.1%, lower than that estimated in the national epidemiologic serosurvey of hepatitis B in China (about 8% among individuals aged 30-59 years).^32^ The low detection rate may lead to a neglectable false-negativity. However, to a certain extent, the false-negativity may underestimate the association strength, as we noticed the HR value was slightly lower than in previous studies. To eliminate concerns, we replicated the analysis in 2 dependent cohorts and a consistent result was obtained. Hepatitis C core antibody was not detected in the study population; however, the prevalence of hepatitis C core antibody was low in China (0.43%).^22^ Hepatitis B surface antigen positivity suggests current infection, and anti-HBc positivity suggests past infection or occult infection. The tissue-based test indicated that HBV replicated in nonliver infection was not released into blood circulation. Further tests involving more nonliver tissues are needed to confirm the HBV DNA–positive rate and the strength of the association between HBV infection and extrahepatic cancer.

## Conclusions

This study suggests that HBV infection is associated with the risk of nonliver cancers, especially digestive system cancers among adults in China. These findings highlight the importance of early screening for digestive system cancers in individuals infected with HBV.

## Notes

Supplement. **eTable 1.** *ICD-10* Codes for Specific Malignant Neoplasms **eTable 2.** Baseline Characteristics of the CKB Participants According to the HBsAg Status **eTable 3.** The Incidence Rates of Cancer in the CKB Cohort **eTable 4.** Adjusted Odds Ratio for HBsAg Status and Stomach Cancer Risk in the Changzhou Nested Case-Control Study **eTable 5.** Distribution of HBV DNA in Stomach Tumor Tissues **eTable 6.** The Expression of Anti-HBc and HBX in Stomach, Pancreatic and Lung Cancer Tissues **eFigure 1.** Results of PCR Tests Performed to Detect HBV DNA and cccDNA in the Tissues of all Stomach Cancer Subjects **eFigure 2**. Anti-HBc and HBx Expression in Tissues of Stomach Cancer, Pancreatic Cancer, or Lung Cancer Using the IHC Test

## References

[1] Liaw YF, Chu CM Hepatitis B virus infection. Lancet. 2009;373(9663):-. doi:10.1016/S0140-6736(09)60207-5 19217993

[2] Arbuthnot P, Kew M Hepatitis B virus and hepatocellular carcinoma. Int J Exp Pathol. 2001;82(2):77-100. doi:10.1111/j.1365-2613.2001.iep178.x 11454100 PMC2517704

[3] Lucifora J, Protzer U Attacking hepatitis B virus cccDNA—the holy grail to hepatitis B cure. J Hepatol. 2016;64(1)(suppl):S41-S48. doi:10.1016/j.jhep.2016.02.009 27084036

[4] Levrero M, Zucman-Rossi J Mechanisms of HBV-induced hepatocellular carcinoma. J Hepatol. 2016;64(1)(suppl):S84-S101. doi:10.1016/j.jhep.2016.02.021 27084040

[5] Dejean A, Lugassy C, Zafrani S, Tiollais P, Brechot C Detection of hepatitis B virus DNA in pancreas, kidney and skin of two human carriers of the virus. J Gen Virol. 1984;65(pt 3):651-655. doi:10.1099/0022-1317-65-3-651 6699625

[6] Mason A, Wick M, White H, Perrillo R Hepatitis B virus replication in diverse cell types during chronic hepatitis B virus infection. Hepatology. 1993;18(4):781-789. doi:10.1002/hep.1840180406 8406351

[7] Chen NL, Bai L, Deng T, Zhang C, Kong QY, Chen H Expression of hepatitis B virus antigen and *Helicobacter pylori* infection in gastric mucosa of patients with chronic liver disease. Hepatobiliary Pancreat Dis Int. 2004;3(2):223-225. 15138114

[8] Iloeje UH, Yang HI, Jen CL, Risk of pancreatic cancer in chronic hepatitis B virus infection: data from the REVEAL-HBV cohort study. Liver Int. 2010;30(3):423-429. doi:10.1111/j.1478-3231.2009.02147.x 19840258

[9] Ulcickas Yood M, Quesenberry CP Jr, Guo D, Incidence of non-Hodgkin’s lymphoma among individuals with chronic hepatitis B virus infection. Hepatology. 2007;46(1):107-112. doi:10.1002/hep.21642 17526021

[10] Andersen ES, Omland LH, Jepsen P, ; DANVIR Cohort Study Risk of all-type cancer, hepatocellular carcinoma, non-Hodgkin lymphoma and pancreatic cancer in patients infected with hepatitis B virus. J Viral Hepat. 2015;22(10):828-834. doi:10.1111/jvh.12391 25650146

[11] Sundquist K, Sundquist J, Ji J Risk of hepatocellular carcinoma and cancers at other sites among patients diagnosed with chronic hepatitis B virus infection in Sweden. J Med Virol. 2014;86(1):18-22. doi:10.1002/jmv.23754 24038002

[12] Kamiza AB, Su FH, Wang WC, Sung FC, Chang SN, Yeh CC Chronic hepatitis infection is associated with extrahepatic cancer development: a nationwide population-based study in Taiwan. BMC Cancer. 2016;16(1):861. doi:10.1186/s12885-016-2918-5 27821099 PMC5100218

[13] Thompson AJ, Nguyen T, Iser D, Serum hepatitis B surface antigen and hepatitis B e antigen titers: disease phase influences correlation with viral load and intrahepatic hepatitis B virus markers. Hepatology. 2010;51(6):1933-1944. doi:10.1002/hep.23571 20512987

[14] Manesis EK, Papatheodoridis GV, Tiniakos DG, Hepatitis B surface antigen: relation to hepatitis B replication parameters in HBeAg-negative chronic hepatitis B. J Hepatol. 2011;55(1):61-68. doi:10.1016/j.jhep.2010.10.027 21145875

[15] Chen Z, Lee L, Chen J, Cohort profile: the Kadoorie Study of Chronic Disease in China (KSCDC). Int J Epidemiol. 2005;34(6):1243-1249. doi:10.1093/ije/dyi174 16131516

[16] Chen Z, Chen J, Collins R, ; China Kadoorie Biobank (CKB) collaborative group China Kadoorie Biobank of 0.5 million people: survey methods, baseline characteristics and long-term follow-up. Int J Epidemiol. 2011;40(6):1652-1666. doi:10.1093/ije/dyr120 22158673 PMC3235021

[17] Chen YS, Chen JG, Zhu J, Zhang YH, Ding LL Long-term survival trends of gastric cancer patients between 1972 and 2011 in Qidong. Chin J Cancer. 2015;34(12):602-607. 26481511 10.1186/s40880-015-0058-y PMC4615360

[18] Chen W, Lu F, Liu SJ, Cancer risk and key components of metabolic syndrome: a population-based prospective cohort study in Chinese. Chin Med J (Engl). 2012;125(3):481-485. 22490407

[19] Laras A, Koskinas J, Dimou E, Kostamena A, Hadziyannis SJ Intrahepatic levels and replicative activity of covalently closed circular hepatitis B virus DNA in chronically infected patients. Hepatology. 2006;44(3):694-702. doi:10.1002/hep.21299 16941694

[20] GenBank overview. National Center for Biotechnology Information website. https://www.ncbi.nlm.nih.gov/genbank/. Accessed May 20, 2019.

[21] Ali A, Abdel-Hafiz H, Suhail M, Hepatitis B virus, HBx mutants and their role in hepatocellular carcinoma. World J Gastroenterol. 2014;20(30):10238-10248. doi:10.3748/wjg.v20.i30.10238 25132741 PMC4130832

[22] Cui Y, Jia J Update on epidemiology of hepatitis B and C in China. J Gastroenterol Hepatol. 2013;28(suppl 1):7-10. doi:10.1111/jgh.12220 23855289

[23] Chen CJ, Yang HI, Su J, ; REVEAL-HBV Study Group Risk of hepatocellular carcinoma across a biological gradient of serum hepatitis B virus DNA level. JAMA. 2006;295(1):65-73. doi:10.1001/jama.295.1.65 16391218

[24] Wright TL, Lau JY Clinical aspects of hepatitis B virus infection. Lancet. 1993;342(8883):1340-1344. doi:10.1016/0140-6736(93)92250-W 7694023

[25] Sundaram V, Kowdley K Management of chronic hepatitis B infection. BMJ. 2015;351:h4263. doi:10.1136/bmj.h4263 26491030

[26] Wei XL, Qiu MZ, Jin Y, Hepatitis B virus infection is associated with gastric cancer in China: an endemic area of both diseases. Br J Cancer. 2015;112(7):1283-1290. doi:10.1038/bjc.2014.406 25695484 PMC4385949

[27] Seeger C, Mason WS Hepatitis B virus biology. Microbiol Mol Biol Rev. 2000;64(1):51-68. doi:10.1128/MMBR.64.1.51-68.2000 10704474 PMC98986

[28] Schieck A, Schulze A, Gähler C, Hepatitis B virus hepatotropism is mediated by specific receptor recognition in the liver and not restricted to susceptible hosts. Hepatology. 2013;58(1):43-53. doi:10.1002/hep.26211 23292963

[29] Wang Y, Zhou H, Zhang L, Prevalence of chronic hepatitis B and status of HBV care among rural women who planned to conceive in China. Sci Rep. 2017;7(1):12090. doi:10.1038/s41598-017-12005-2 28935971 PMC5608955

[30] Huang P, Zhu LG, Zhu YF, Seroepidemiology of hepatitis B virus infection and impact of vaccination. World J Gastroenterol. 2015;21(25):7842-7850. doi:10.3748/wjg.v21.i25.7842 26167084 PMC4491971

[31] Ko YC, Yen YY, Yeh SM, Lan SJ Female to male transmission of hepatitis B virus between Chinese spouses. J Med Virol. 1989;27(2):142-144. doi:10.1002/jmv.1890270214 2921601

[32] Liang X, Bi S, Yang W, Epidemiological serosurvey of hepatitis B in China—declining HBV prevalence due to hepatitis B vaccination. Vaccine. 2009;27(47):6550-6557. doi:10.1016/j.vaccine.2009.08.048 19729084

