# Supplementary material for: Associations Between Hepatitis B Virus Infection and Risk of All Cancer Types
Source: JAMA Netw Open. 2019 Jun 14;2(6):e195718. doi: 10.1001/jamanetworkopen.2019.5718 (PMC6575146; doi:10.1001/jamanetworkopen.2019.5718)
Supplement: Supplement. — eTable 1. ICD-10 Codes for Specific Malignant Neoplasms eTable 2. Baseline Characteristics of the CKB Participants According to the HBsAg Status eTable 3. The Incidence Rates of Cancer in the CKB Cohort eTable 4. Adjusted Odds Ratio for HBsAg Status and Stomach Cancer Risk in the Changzhou Nested Case-Control Study eTable 5. Distribution of HBV DNA in Stomach Tumor Tissues eTable 6. The Expression of Anti-HBc and HBX in Stomach, Pancreatic and Lung Cancer Tissues eFigure 1. Results of PCR Tests Performed to Detect HBV DNA and cccDNA in the Tissues of all Stomach Cancer Subjects eFigure 2. Anti-HBc and HBx Expression in Tissues of Stomach Cancer, Pancreatic Cancer, or Lung Cancer Using the IHC Test [file jamanetwopen-2-e195718-s001.pdf]

Supplementary Online Content

Song C, Lv J, Liu Y, et al; China Kadoorie Biobank Collaborative Group. Associations between hepatitis B virus infection and risk of all cancer types. *JAMA Netw Open.* 2019;2(6):e195718. doi:10.1001/jamanetworkopen.2019.5718

eTable 1. *ICD-10* Codes for Specific Malignant Neoplasms
eTable 2. Baseline Characteristics of the CKB Participants According to the HBsAg Status
eTable 3. The Incidence Rates of Cancer in the CKB Cohort
eTable 4. Adjusted Odds Ratio for HBsAg Status and Stomach Cancer Risk in the Changzhou Nested Case-Control Study
eTable 5. Distribution of HBV DNA in Stomach Tumor Tissues
eTable 6. The Expression of Anti-HBc and HBX in Stomach, Pancreatic and Lung Cancer Tissues
eFigure 1. Results of PCR Tests Performed to Detect HBV DNA and cccDNA in the Tissues of all Stomach Cancer Subjects
eFigure 2. Anti-HBc and HBx Expression in Tissues of Stomach Cancer, Pancreatic Cancer, or Lung Cancer Using the IHC Test

This supplementary material has been provided by the authors to give readers additional information about their work.

**eTable 1.** *ICD-10* Codes for Specific Malignant Neoplasms		
Category	*ICD-10*	Abbreviation
All cancer	C00-C97	—
Lip, oral cavity and pharynx cancer	C00-C14	Oral cavity
Esophagus cancer	C15	Esophagus
Stomach cancer	C16	Stomach
Colon, Rectosigmoid junction and Rectum	C18-C20	Colorectum
Liver cancer	C22	Liver
Pancreas cancer	C25	Pancreas
Trachea, Bronchus and Lung	C33-C34	Lung
Breast cancer	C50	Breast
Cervix uteri cancer	C53	Cervix
Endometrium cancer	C54.1	Endometrium
Ovary cancer	C56	Ovary
Prostate cancer	C61	Prostate
Bladder cancer	C67	Bladder
Lymphoma	C81-C85	Lymphoma
Leukemia	C91-C95	Leukemia

ICD-10, *International Classification of Diseases-10*.

© 2019 Song C et al. *JAMA Network Open.*

eTable 2. Baseline Characteristics of the CKB Participants According to the HBsAg Status

Characteristic	All Participants (n = 496,732)	HBsAg-positive (n = 15,355)
Age, n (%)		
<50 years	224,086 (45.1)	8,311 (3.7)
⩾50 years	272,646 (54.9)	7,044 (2.6)
Sex, n (%)		
Women	293,248 (59.0)	8,300 (2.8)
Men	203,484 (41.0)	7,055 (3.5)
Area, n (%)		
Urban	221,236 (44.5)	7,848 (3.5)
Rural	275,496 (55.5)	7,507 (2.7)
Region, n (%)		
Qingdao (Urban)	34,499 (6.9)	1,030 (3.0)
Harbin (Urban)	56,708 (11.4)	1,134 (2.0)
Haikou (Urban)	29,508 (5.9)	1,543 (5.2)
Suzhou (Urban)	51,511 (10.4)	2,200 (4.3)
Liuzhou (Urban)	49,010 (9.9)	1,941 (4.0)
Pengzhou (Rural)	51,797 (10.4)	949 (1.8)
Tianshui (Rural)	48,802 (9.8)	914 (1.9)
Huixian (Rural)	62,464 (12.6)	2,152 (3.4)
Tongxiang (Rural)	53,353 (10.7)	1,876 (3.5)
Liuyang (Rural)	59,080 (11.9)	1,616 (2.7)
Married, n (%)		
No	46,715 (9.4)	1,247 (2.7)
Yes	450,017 (90.6)	14,108 (3.1)
Educational level, n (%)		
No formal education	90,335 (18.2)	2,405 (2.6)
Primary school	159,951 (32.2)	4,706 (2.9)
Middle or high school	217,092 (43.7)	7,448 (3.4)
College or university	29,354 (5.91)	796 (2.7)
Household income, n (%)		
<10,000 yuan/yr	139,457 (28.1)	3,670 (2.6)
10,000—19,999 yuan/yr	144,915 (29.2)	4,541 (3.1)
20,000—34,999 yuan/yr	122,513 (24.7)	4,075 (3.3)
⩾35,000 yuan/yr	89,847 (18.1)	3,069 (3.4)
Family history of cancer, n		
No	398,999 (82.7)	11,964 (3.0)
Yes	83,405 (17.3)	3,025 (3.6)
Regular smoking, n (%)		
Never	336,462 (67.7)	9,988 (3.0)
Current/Previous	160,270 (32.3)	5,367 (3.3)
Regular alcohol intake, n		
Never	414,669 (83.5)	12,714 (3.1)
Current/Previous	82,063 (16.5)	2,641 (3.2)
Body mass index, n (%)		
<24.0	278,450 (56.1)	8,961 (3.2)
24.0-27.9	165,359 (33.3)	4,854 (2.9)
⩾28.0	52,921 (10.7)	1,540 (2.9)

© 2019 Song C et al. *JAMA Network Open.*

At exchange rate as of June 2017, 1 yuan is approximately equal to 0.15 U.S. dollars.

© 2019 Song C et al. *JAMA Network Open.*

eTable 3. The Incidence Rates of Cancer in the CKB Cohort

Rank	Type	No. of cases	No. of person-years	Crude-incidence ($1/10^5$)	Standardized-incidence ($1/10^5$)*
	All	20,891	4,430,988	471.48	422.21
1	Liver	1,775	4,430,988	40.06	40.28
2	Lung	3,847	4,430,988	86.82	78.23
3	Stomach	2,159	4,430,988	48.73	47.08
4	Colorectum	2,139	4,430,988	48.27	40.17
5	Breast	1,694	2,642,521	64.11	52.04
6	Esophagus	1,589	4,430,988	35.86	42.13
7	Oral cavity	416	4,430,988	9.39	8.61
8	Lymphoma	369	4,430,988	8.33	7.58
9	Pancreas	505	4,430,988	11.4	9.4
10	Cervix	699	2,642,521	26.45	25.92
11	Leukemia	430	4,430,988	9.7	8.73
12	Bladder	344	4,430,988	7.76	6.81
13	Ovary	263	2,642,521	9.95	8.91
14	Endometrium	273	2,642,521	10.33	8.35
15	Prostate	254	1,788,466	14.2	11.1

* Standardized by age, sex and region based on the 2010 Chinese census population.

© 2019 Song C et al. *JAMA Network Open.*

eTable 4. Adjusted Odds Ratio for HBsAg Status and Stomach Cancer Risk in the Changzhou Nested Case-Control Study

Variable	Stomach cancer cases	Healthy controls	OR (95% CI)	*P*
	N (%)	N (%)		
HBsAg				
Negative	94 (79.7)	412 (87.3)	1.00	0.04
Positive	24 (20.3)	60 (12.7)	1.76 (1.04-2.98)	

Logistic regression analyses were adjusted for age, sex, education level, household income, marital status, smoking status, alcohol consumption, family cancer history, and BMI.

© 2019 Song C et al. *JAMA Network Open.*

eTable 5. Distribution of HBV DNA in Stomach Tumor Tissues

	Jiangsu (n = 47)			Zhejiang (n = 50)			All patients (n = 97)		
Variable	HBV DNA positive	HBV DNA negative		HBV DNA positive	HBV DNA negative		HBV DNA positive	HBV DNA negative	*P*
	N (%)	N (%)		N (%)	N (%)		N (%)	N (%)	
All patients	12 (25.5)	35 (74.5)		19 (38.0)	31 (62.0)		31 (32.0)	66 (68.0)	-
HBV Status in serum									
anti-HBc (+)	12 (54.5)	10 (45.5)		19 (54.3)	16 (45.7)		31 (54.4)	26 (45.6)	-
anti-HBc (-)	0 (0)	25 (100.0)		0 (0)	15 (100.0)		0 (0)	40 (100.0)	
Gender									
Male	10 (27.8)	26 (72.2)		14 (37.8)	23 (62.2)		24 (32.9)	49 (67.2)	0.74
Female	2 (18.2)	9 (81.8)		5 (38.5)	8 (61.5)		7 (21.2)	17 (70.8)	
Age									
<60 years	1 (11.1)	8 (88.9)		8 (36.4)	14 (63.6)		9 (29.0)	22 (70.1)	0.67
⩾60 years	11 (28.9)	27 (71.1)		11 (39.3)	17 (60.7)		22 (33.3)	44 (66.7)	

P for chi-square test for differences between subgroups with different HBV, sex and age statuses.

© 2019 Song C et al. *JAMA Network Open.*

eTable 6. The Expression of Anti-HBc and HBX in Stomach, Pancreatic and Lung Cancer Tissues

ID	Sex	Age at diagnosis	Diagnosis	Sero-HBsAg	Sero-anti-HBc	Anti-HBc in tissue	HBX in tissue
1	Male	67	Stomach cancer	-	+	+	+
2	Male	75	Stomach cancer	+	+	+	+
3	Male	59	Stomach cancer	-	+	+	+
4	Male	65	Stomach cancer	-	+	+	+
5	Male	61	Stomach cancer	-	+	+	+
6	Female	50	Stomach cancer	-	+	+	+
7	Male	66	Stomach cancer	-	+	+	+
8	Female	64	Stomach cancer	-	+	+	+
9	Male	58	Pancreatic cancer	-	+	+	+
10	Female	62	Pancreatic cancer	+	+	+	+
11	Male	53	Pancreatic cancer	+	+	+	+
12	Female	61	Pancreatic cancer	+	+	+	+
13	Male	64	Pancreatic cancer	+	+	+	+
14	Male	73	Pancreatic cancer	-	+	+	+
15	Female	61	Pancreatic cancer	-	+	+	+
16	Male	52	Pancreatic cancer	+	+	+	+
17	Female	67	Pancreatic cancer	+	+	+	+
18	Male	86	Pancreatic cancer	+	+	+	+
19	Female	52	Lung cancer	-	+	-	-
20	Male	63	Lung cancer	+	+	-	-
21	Female	64	Lung cancer	-	+	-	-
22	Female	69	Lung cancer	-	+	-	-
23	Female	65	Lung cancer	-	+	-	-
24	Male	66	Lung cancer	-	+	-	-
25	Female	66	Lung cancer	-	+	-	-
26	Male	56	Lung cancer	-	+	-	-
27	Female	43	Lung cancer	+	+	-	-

"+" represents positive expression; "-" represents negative expression.

© 2019 Song C et al. *JAMA Network Open.*

eFigure 1. Results of PCR Tests Performed to Detect HBV DNA and cccDNA in the Tissues of all Stomach Cancer Subjects

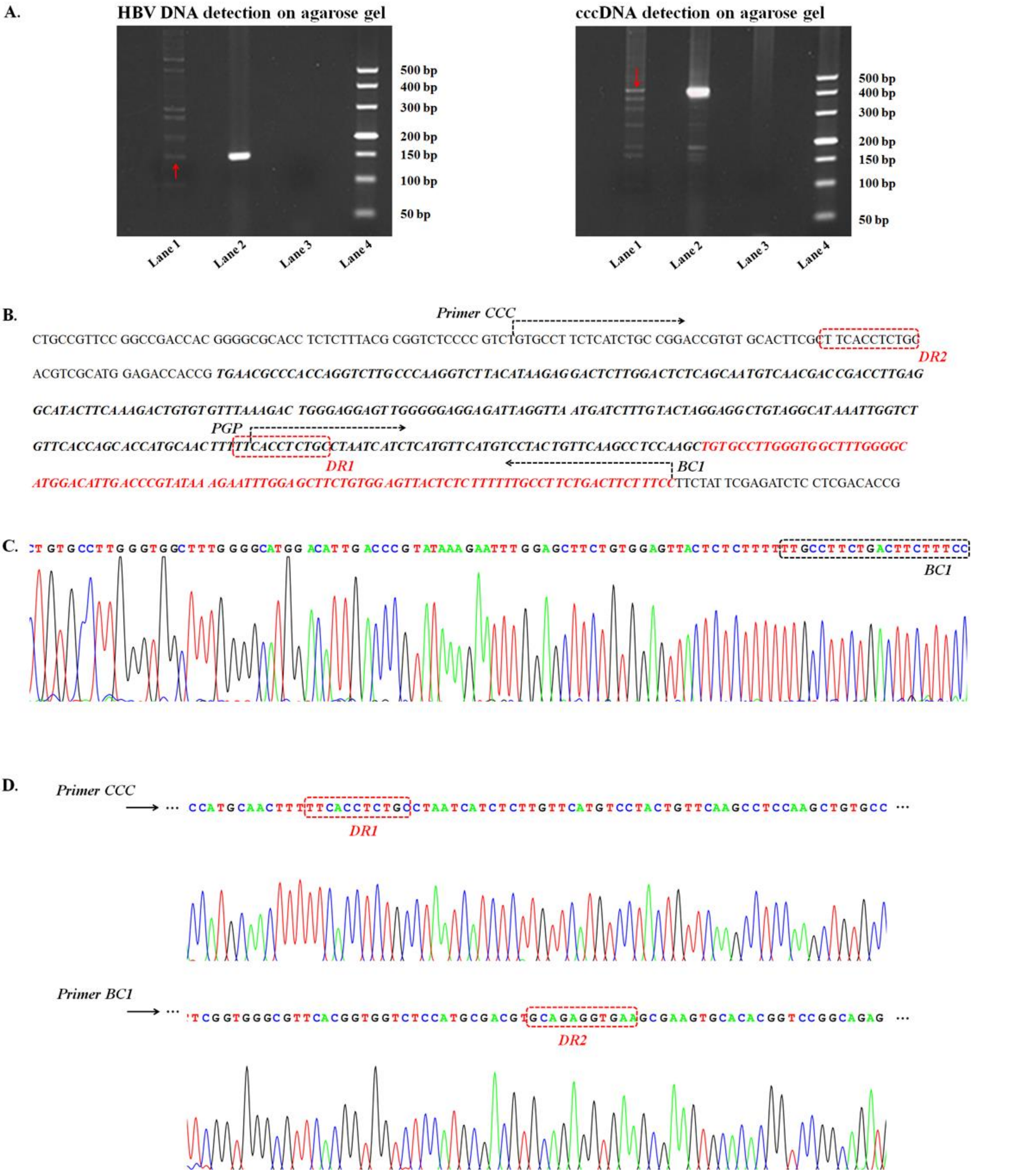

© 2019 Song C et al. *JAMA Network Open.*

eFigure 2. Anti-HBc and HBx Expression in Tissues of Stomach Cancer, Pancreatic Cancer, or Lung Cancer Using the IHC Test

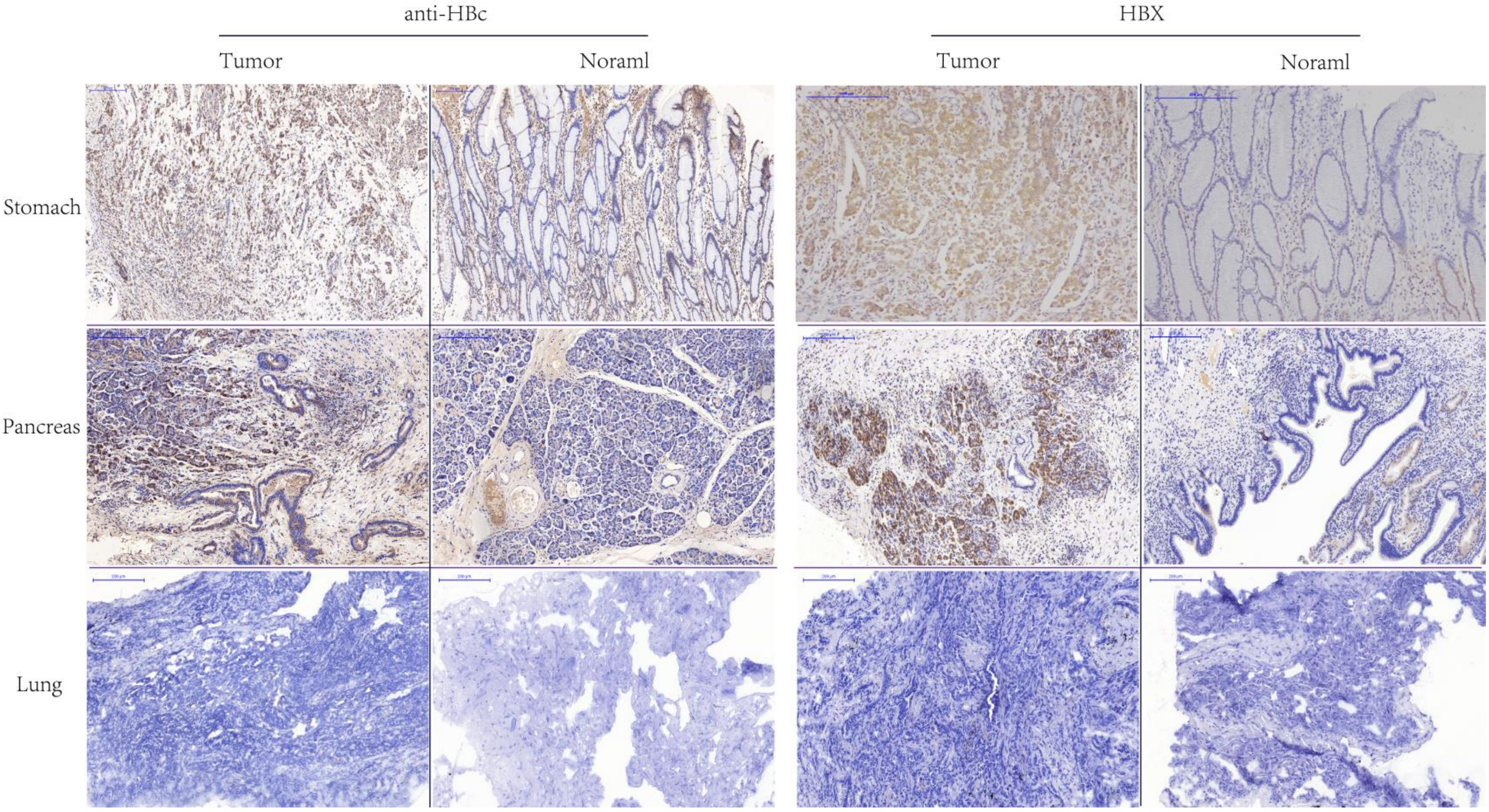

© 2019 Song C et al. *JAMA Network Open.*
